# In-vitro pH-responsive release of imatinib from iron-supplement coated anatase TiO_2_ nanoparticles

**DOI:** 10.1038/s41598-022-08090-7

**Published:** 2022-03-17

**Authors:** Shilpy Bhullar, Navdeep Goyal, Shikha Gupta

**Affiliations:** 1 Department of Physics, Centre of Advanced Study in Physics, Panjab University, Chandigarh, 160014 India; 2 Department of Physics, Goswami Ganesh Dutta Sanatan Dharma College, Sector-32C, Chandigarh, 160032 India

**Keywords:** Magnetic properties and materials, Nanoparticles, Structural properties, Synthesis and processing, Drug delivery, Nanotechnology in cancer, Materials science, Nanoscience and technology

## Abstract

Targeted drug delivery is one such precision method of delivering medication inside the human body which can vanquish all the limitations of the conventional chemotherapeutic techniques. In the present study, two types of nanoparticles (NPs) were chosen for the in-vitro pH-responsive release study of the drug, Imatinib, namely anatase Titanium Dioxide nanoparticles (TiO_2_ NPs) and iron-capped TiO_2_ NPs, designated as Fe@TiO_2_ NPs. The novelty of this work lies behind the use of commercially available iron supplement ‘Autrin’ meant for human consumption, as the material to coat the TiO_2_ NPs to synthesize Fe@TiO_2_ NPs. The synthesized NPs were analyzed by XRD, HR‐TEM, SAED, EDX and VSM. UV–Vis spectroscopy was performed for absorption studies. Fe@TiO_2_ NPs showed superparamagnetic behavior and thus they are able to ensure the facile transfer of Imatinib via external magnetic fields. The results obtained from in-vitro drug release studies depicted that both TiO_2_ NPs and Fe@TiO_2_ NPs showed a controlled pH-sensitive delivery of the loaded Imatinib molecules. Moreover, both types of NPs do not result in the formation of ROS under human physiological conditions. These results can lay the foundation to the development of efficacious targeted drug delivery systems in the healthcare sector.

## Introduction

Today, we are witnessing a global pandemic whose impact has been devastatingly pervading. Not to mention, many new diseases caused by fungi and other microbes are discovered each year. What worsens the situation is our inability to treat life-threatening diseases like cancer which have been known to mankind for quite a long time. Genetic mutations cause many carcinogenic cells to acquire resistance against many drugs thereby convoluting the treatment provided^1^. There is no denying the fact that today we need smarter tools and smarter medicines to provide advanced medical treatments and to combat the deadly diseases. Nanotechnology is the future of mankind. This technology of manipulating the matter at nano scales to obtain remarkably novel materials with unique properties has huge potential in the field of healthcare.

Radiotherapy, surgery and chemotherapy are the major anti-cancer therapies undertaken. But, the non-specific targeting of cancer cells have made these approaches ineffective in a number of patients as they affect the healthy tissues as well. Chemo drugs used in cancer treatment often lack the target-specificity and many times, a combination of chemo drugs is used to ascertain complete treatment. However, not all medicines work in a similar manner and they possess their own side-effects as well. The non-directionality of these drugs result in their action at undesirable sites too. To cope with this, higher doses of drugs are administered which generates major health concerns. Here, nanotechnology can come to the rescue. Nanoparticles (NPs) are the new era tools which can be used to load drugs onto them and ensure targeted drug delivery. This is a method of delivering medications inside the human body by achieving maximum concentration at the target locations and the least concentration around the normal tissues. This confirms the least drug wastage in the other regions of the body and maximum dosage released at the site of action^2^.

The state of the art in medical research involves using the NPs as drug carriers and following different approaches to achieve targeted drug delivery. One of the approaches involves conjugating the drug to a cell-specific ligand or tissue to have active targeted drug delivery, whereas, the other involves encapsulation of NPs or macromolecules with a therapeutic agent which passively reaches the target through Enhanced Permeability and Retention (EPR) effect. Liposomes and other biologically modified NPs are often used for the efficient delivery of lipophilic drugs which dissolve through the outer lipid membrane of the NPs right into the target cells via lipid-lipid exchange^3^. Infact, NPs have been reported to cross the Blood–Brain Barrier (BBB) and thus, they are favorable in treating difficult-to-treat tumors. Most of the tumor cells are recognized by the over-expression of the biomarker folate receptor. Therefore, many NPs to be used as drug-carriers are conjugated with folic acid as they will target those locations having the folate receptors present in abundance^4^. The majority of chemo drugs are expected to be released under acidic environments as the pH around malignant tumors is generally low. The drug bearing NPs must be synthesized in such a way that they promote the drug-disintegration under lower pH levels rather than under normal pH levels^5^. We can infer that all these means discussed hereby provide a directional locomotion to the NPs. Likewise, magnetic fields can also be used to drive the magnetic NPs to reach the target sites. For this purpose, either magnetic NPs can be used directly or non-magnetic NPs with some other benefits can be modified magnetically. This modification can be done by either doping with magnetic material or making a nanocomposite or employing a core–shell formulation with magnetic material as a core or as a shell^6^. This current study involves using TiO_2_ NPs as the base material and these were coated with iron salt to provide magnetism to the otherwise non-magnetic TiO_2_ NPs. Thus, magnetic fields can provide the desired driving force to these NPs to reach the desired destination in-vivo. However, this experimentation is beyond the scope of the present study. The research undertaken in this paper involves the encapsulation of the resulting core–shell complex with a chemo drug Imatinib to study the drug-release behavior under different pH levels in-vitro.

Imatinib is a chemotherapeutic drug used to treat leukemia and gastrointestinal stromal tumours. It is a class of tyrosine kinase inhibitors. Tyrosine kinases are a group of enzymes which are responsible for many key events in the human body. They are involved in the transfer of extracellular signals via cell membrane to the cytoplasm and in many cases, to the nucleus too. In the nucleus, these control the cell-cycle, induce the mitosis in the cell and are responsible for cell growth and reproduction. Sometimes, a certain mutation can cause these tyrosine kinases to be constitutively active, thereby resulting in unregulated cell divisions. This occurrence is often related to several cancers. The drug Imatinib is capable of inhibiting their activity and is therefore employed for the treatment of cancers caused due to uncontrolled cell growth^7^. The therapeutic efficacy of the drugs depend upon their interaction with subcellular components. These targeted drugs alter the molecular and metabolic functions of the subcellular entities present to enhance the therapeutic efficiency of the drugs on the targeted site. Among all the cellular and subcellular organelles, mitochondria plays a key role in various cellular functions including cell survival, ROS stabilization and regulating cell death. The major indicators of the cells which turn malignant are significant metabolic imbalance and increased resistance to cell death, both of which are regulated by mitochondria. These are the mediators of life and death of cells. Thus, mitochondrial permeability is the key regulator for attaining death of cancerous cells. Through various researches in recent years, it has been observed that the mitochondrial dysfunction altering the apoptosis mechanism is mainly responsible for transforming normal cells into carcinogenic cells. Any abnormality in the functioning of mitochondria modulates the energy metabolism and electron transport chain of the cancerous cells. Dysfunctional mitochondria promotes cancer cell invasion, cancer survival and anticancer drug resistance. Nowadays, mitochondria-targeted drug delivery systems are being mechanised making mitochondria—the prime target of new anticancer drugs^8^. Chen et al.^9^ reported that Imatinib induces mitochondria-assisted apoptosis and restricts the invasion of pigmented villonodular synovitis cells. With the approval of Imatinib by the Food and Drug Administration (FDA) in 2001, it has been utilized to treat chronic and acute myeloid leukaemia since then. Studies have proved that mitochondrial metabolism can be treated as a potential therapeutic target in treating myeloid leukaemia^10^.

**Figure 1:**
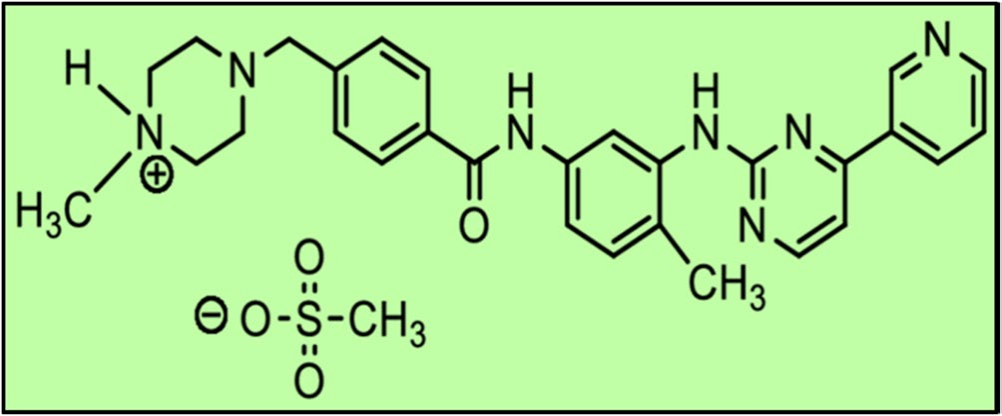
Chemical structure of Imatinib mesylate.

Imatinib is an antineoplastic agent which induces apoptosis in cells overexpressing certain oncoproteins and thus impedes the build-up of such cells. This small molecule kinase inhibitor is orally administered in the form of a tablet. Each tablet contains Imatinib Mesylate equivalent to 100 mg or 400 mg of the Imatinib free base. The IUPAC name of Imatinib Mesylate is 4-4[(4-methyl-1-piperazinyl)methyl]-N-[4-methyl-3-[[4-(3-pyridinyl)-2-pyrimidinyl]amino]-phenyl]—benzamide mono methane sulfonate. Its molecular formula is C_29_H_31_N_7_O.CH_4_SO_3_ and its molecular weight is 589.7. Its chemical structure is shown in Fig. 1. Imatinib Mesylate is very much dissolvable in aqueous buffers with pH ≤ 5.5 and is partially soluble to insoluble in neutral and alkaline buffers respectively. Its mean absolute bioavailability is 98% and the elimination half-lives of Imatinib and its major metabolites are 18 h and 40 h respectively. 68% of the drug is excreted through feces and 13% via urine within 7 days^11^.

The toxicity aspect of the NPs must be taken into account when utilizing them as drug-transporters within the human body. The metal oxide NPs are usually associated with generation of Reactive Oxygen Species (ROS) which are highly reactive oxygen radicals leading to cell death. TiO_2_ NPs are known to be excellent photocatalysts as they generate ROS when irradiated with ultraviolet light. This utility of TiO_2_ NPs is being studied effectively for Photodynamic Therapy these days^12^. But this application of TiO_2_ NPs might prove to be perilous for drug-delivery application when these NPs would be sent inside the human body. One cannot take the risk of doing more harm than good if these NPs are supposed to generate ROS under human physiological conditions while transporting drugs. Infact, when the antioxidant defense of the cells is not strong enough to protect themselves from any type of ROS generation taking place, this sort of imbalance generally results in onset of cancer among those otherwise healthy cells. However, ROS are not all bad, they have several important benefits as well. They are important messengers in cell signaling pathways and thus contribute in controlling normal functions of the cells. Intracellular ROS generation is particularly beneficial for killing bacteria by macrophages where antioxidants might interfere with this process and worsen the condition^13^. In a nutshell, ROS might have their own uses but they are not required while delivering drugs under normal cell settings. Thus, it is imperative to check for any ROS formation by the NPs during drug-delivery applications.

The NPs possessing antioxidant properties can solve the issue of combating ROS formation. Nowadays, the NPs synthesized specifically to exhibit antioxidant properties are termed as nano-antioxidants. The extended surface area of the nano-antioxidants not only accelerates the radical scavenging performance but also provides greater opportunities for functionalization and other surface modifications to ensure targeted delivery^14^. Most of the green-synthesized TiO_2_ NPs are also known to exhibit antioxidant properties^15,16^. Thus, by changing different synthesis parameters, antioxidant behaviour of the TiO_2_ NPs can be modified.

The present study involves studying the in-vitro release behavior under different pH environments of (1) the drug loaded onto TiO_2_ NPs and (2) the drug loaded onto iron-coated TiO_2_ NPs (Fe@TiO_2_ NPs). The TiO_2_ NPs were chemically synthesized via sol–gel technique. In order to synthesize magnetically guided NPs, there was a need for magnetic modification of the synthesized TiO_2_ NPs. This was done by coating the NPs with a magnetic material. Instead of using chemicals for this purpose, a commercially available iron supplement, Autrin, was chosen. As these iron supplements are designed for human consumption, these are suitable for enhancing the biocompatibility of the core NPs. Moreover, iron tablets are often conjugated with folic acid, otherwise, the tablet might cause stomach issues. The carcinogenic cells are frequently associated with acidic pH and folate receptors. Thus, an iron salt along with folic acid is advantageous to be used in nanomedicines meant for cancer treatment. Additionally, iron supplements lower the levels of oxidative stress. Iron deficiency is also associated with the increase of oxidative stress among patients. It has been tested that oxidative stress diminishes once the iron stores of the body are restored. Iron supplementation is recommended to recover the impaired antioxidant defense system of the body^17^. The folic acid present in the autrin tablets used for the present study is also known to improve the biomarkers associated with the antioxidant defense system of the body^18^. The vitamin B_12_ also holds potential antioxidant properties such as direct scavenging of ROS, preserving glutathione which indirectly results in ROS scavenging and modulating cytokine and growth factor production which protects from oxidative stress induced due to immune response^19^. Thus, the coating of autrin tablets over TiO_2_ NPs provides the necessary protection from any ROS generation but still to ensure the biosafety of the NPs synthesized, the ROS detection test has also been performed. This test has been performed using Nitro Blue Tetrazolium chloride (NBT) which forms a yellow or slightly brown coloured solution. In the presence of oxygen radicals, NBT degrades to blue coloured insoluble formazan. Thus, it is a colourimetric assay to detect ROS formation where any colour change from yellow to blue dictates the conversion of NBT into formazan thereby indicating the presence of superoxide anion radicals. Due to this degradation, the absorption band associated with NBT at 259 nm also decreases. Thus, on adding NPs to the NBT solution and carefully recording the absorbance of the solution at different intervals of time, any decline in the absorption peak would verify the existence of ROS in the sample thereby claiming the ROS generation behaviour of the NPs^20^.

## Experimental section

### Materials

Titanium Tetra Isopropoxide (TTIP), Ethanol, Nitric Acid, Nitro Blue Tetrazolium Chloride (NBT), Phosphate Buffer Saline (PBS) solution of pH 7.4, Acetate Buffer solution of pH 4.4 and Borate Buffer solution of pH 9.0 were purchased from Sigma Aldrich, India. DI water was obtained from the Fractional Distillation unit installed in the Department of Chemistry, Goswami Ganesh Dutta Sanatan Dharma College, Sector-32C, Chandigarh. Imatinib manufactured by Cipla, India and Autrin capsules by Pfizer, Ltd. were purchased from a licensed pharmacist, Chandigarh. Dialysis tubing of 8 kDa was purchased from Himedia. All the purchased materials were used as such without any modification. A magnetic stirrer with hotplate and muffle furnace by INSIF, India were used for stirring and calcination purposes. An incubator capable of establishing human physiological conditions was used for ROS detection study, provided by the Department of Biotechnology, Goswami Ganesh Dutta Sanatan Dharma College, Sector-32C, Chandigarh, India.

### Methods

Sol–gel method was utilized to synthesize the anatase TiO_2_ NPs^21^. A stoichiometric quantity of TTIP was added to 50 ml ethanol and stirred for half an hour. To this solution, 40 ml DI water and a regulated amount of nitric acid were added dropwise to obtain the desired pH. The final solution was stirred for an hour and was then subjected to heating at 60 °C. The onset of heating led to the formation of gel-like substance. The sample so obtained was further calcined at 400 °C to obtain the desired anatase phase. To synthesize Fe@TiO_2_ NPs, Autrin capsules were used. Each capsule of Autrin contains 900 mg Ferrous Fumarate, 1.5 mg Folic Acid and 15 μg Vitamin B_12_. The concentration of Ferrous Fumarate salt present in these capsules is equivalent to 98.6 mg of elemental iron. This salt offers higher absorption inside the human body. For the iron coated TiO_2_ NPs, the procedure mentioned by Mehr et al.^22^ was followed. An optimal amount of ferrous fumarate tablet was chosen in order to have a 1:1 core–shell ratio. The intended amount of the iron tablet was crushed and added to 250 ml DI water. After the salt dissolved completely, the TiO_2_ NPs were added to the solution and the mixture was kept on stirring for 3 h. Next, 1 M NaOH solution was added dropwise and again the solution was left for 5 h on continuous stirring. Finally, the solution was refluxed at 90 °C. The resulting solution was centrifuged, washed with DI water to remove any impurities and then again calcined at 400 °C. The characteristic white color of TiO_2_ NPs was changed into a brown color thereby confirming the coating of Fe^2+^ onto them.

### Characterization

NPs have been characterized for their physical, morphological and magnetic properties. X-ray diffraction (XRD) was performed by a Panalytical X'Pert Pro equipped with x'Celerator solid state detector manufactured by Panalytical, Netherlands. For XRD, the powder sample is firmly smeared on a slide that has been carefully placed on the sampling stage. This characterization was done at the Sophisticated Analytical Instrumentation Facility (SAIF), Panjab University, Chandigarh, India. High-Resolution Transmission Electron Microscopy (HRTEM), Selective Area Electron Diffraction (SAED) and Energy Dispersive Spectroscopy (EDS) were performed using Jeol 2100 Plus, Japan. Here, the sample was dispersed in ethanol and soaked for one hour. Then a drop of the NPs solution was placed onto the copper grid which was further air-dried at room temperature and after 30 min was used for imaging. These features were performed at CIL, Panjab University, Chandigarh, India. The vibrating sample magnetometer (VSM) used in the study was designed by MicroSense, USA. Samples were analyzed at room temperature (300 K). They were firmly placed and patted to form a thin layer on the sample holder. The instrument was provided by the Department of Chemistry, Guru Nanak Dev University, India. The UV–VIS-NIR Lambda 750 spectrophotometer from Perkin Elmer, USA was used to measure UV absorption spectroscopy. A dispersion of NPs was made in the solvent. The baseline correction of the instrument was done followed by blanking using the reference sample in both the cuvettes. The reference sample simply consisted of the solvent used to disperse the NPs. Finally, the absorption data was collected by placing the cuvette containing solvent dispersed NPs in the sample holder and the cuvette containing only solvent in the reference holder.

### Drug encapsulation

After synthesizing the samples, the next task was to encapsulate them with the drug. To realize this, the procedure followed by Akram et al.^23^ was opted for with minor modification. A fixed quantity of the drug was mixed in 40 ml DI water to which 500 mg of the synthesized NPs were added. The solution was stirred for 2 days and then kept for a day. It was then centrifuged and filtered out. Thus, we obtained two samples, one having the drug loaded onto TiO_2_ NPs (referred to as Drug/TiO_2_ NPs) and the other having the drug loaded onto Fe@TiO_2_ NPs (referred to as Drug/Fe@TiO_2_ NPs). To measure the amount of drug in the obtained yield, the absorbance of the filtrate was measured at 254 nm. The amount of the drug which is successfully trapped onto the NPs is determined by % encapsulation efficiency, which came out to be 31.2% for Drug/TiO_2_ NPs and 29.7% for Drug/Fe@TiO_2_ NPs as calculated using eq.(1). % drug loading reflects the mass-ratio of the drug to the nanomedicine which has been synthesized by conjugating the drug to a fixed quantity of NPs. In the current study, it came out to be 43.3% for Drug/TiO_2_ NPs which means that every 1 gm of the loaded NPs contain 0.43 gm of the drug. % drug loading came out to be 40.8% for Drug/Fe@TiO_2_ NPs. It was calculated by finding the concentration of the drug in the loaded NPs considered for the study divided by the quantity of the NPs taken and then multiplied by 100 as per eq.(2).

1 $${\text{Encapsulation Efficiency}}\;{(}\% {)} = ({\text{W}}_{{\text{Drug on the NPs}}} {\text{/W}}_{{\text{Total drug used}}} ) \times {1}00$$

2 $${\text{Drug Loading}}\;{(}\% {)} = ({\text{Concentration of the drug in the NPs/Weight of the loaded NPs considered for the study}}) \times {1}00$$

### In-vitro drug release studies

pH-dependent release behavior at three different pH levels was investigated. The drug-loaded NPs were inserted into a dialysis tube having a Molecular Weight Cut Off (MWCO) of 8 kDa. In total, 6 sets were prepared. 3 sets of dialysis tubes contained Imatinib-loaded TiO_2_ NPs (Drug/TiO_2_ NPs) and the other 3 sets of the dialysis tube contained Imatinib-loaded Fe@TiO_2_ NPs (Drug/Fe@TiO_2_ NPs). To mimic a neutral pH environment, 2 dialysis tubes containing Drug/TiO_2_ NPs and Drug/Fe@TiO_2_ NPs were dipped into 500 ml of 0.1 M Phosphate buffer solution (pH 7.4) separately. Similarly, for the in-vitro study under an acidic environment, 500 ml of 0.1 M Acetate buffer solution (pH 4.4) was used and likewise, 500 ml of 0.1 M Borate buffer solution (pH 9.0) was used to achieve basicity. The dialysis tubes were hung with the help of clips in their respective beakers containing solutions to imitate different pH environments. The entire setup was stirred at 37 °C. After a fixed interval of time, 3 ml of the dialysate was withdrawn from each of the beakers for monitoring the absorbance of the Imatinib molecules released into the respective buffer at 254 nm with the help of UV–Visible Spectrophotometer. The withdrawn amount of the dialysate was replaced with an equal quantity of the fresh buffer solution to avoid the sink conditions.

### ROS detection test using NBT

NBT solution was prepared by the protocol followed by Kumar et al.^24^ in their study with a minor change. The solution needs to be prepared in an amber coloured bottle. Briefly, 0.1 g NBT was dissolved in 50 mM of Phosphate Buffer Saline (PBS) solution to make a final solution of 20 ml. To this quantity, 5 mg each of TiO_2_ NPs and Fe@TiO_2_ NPs were added separately. These samples were then placed in a laboratory incubator maintaining human physiological conditions and their absorbance was studied at different intervals of time upto 48 h. Any drop in the absorbance band of NBT would suggest the generation of ROS.

## Results and discussion

**Figure 2:**
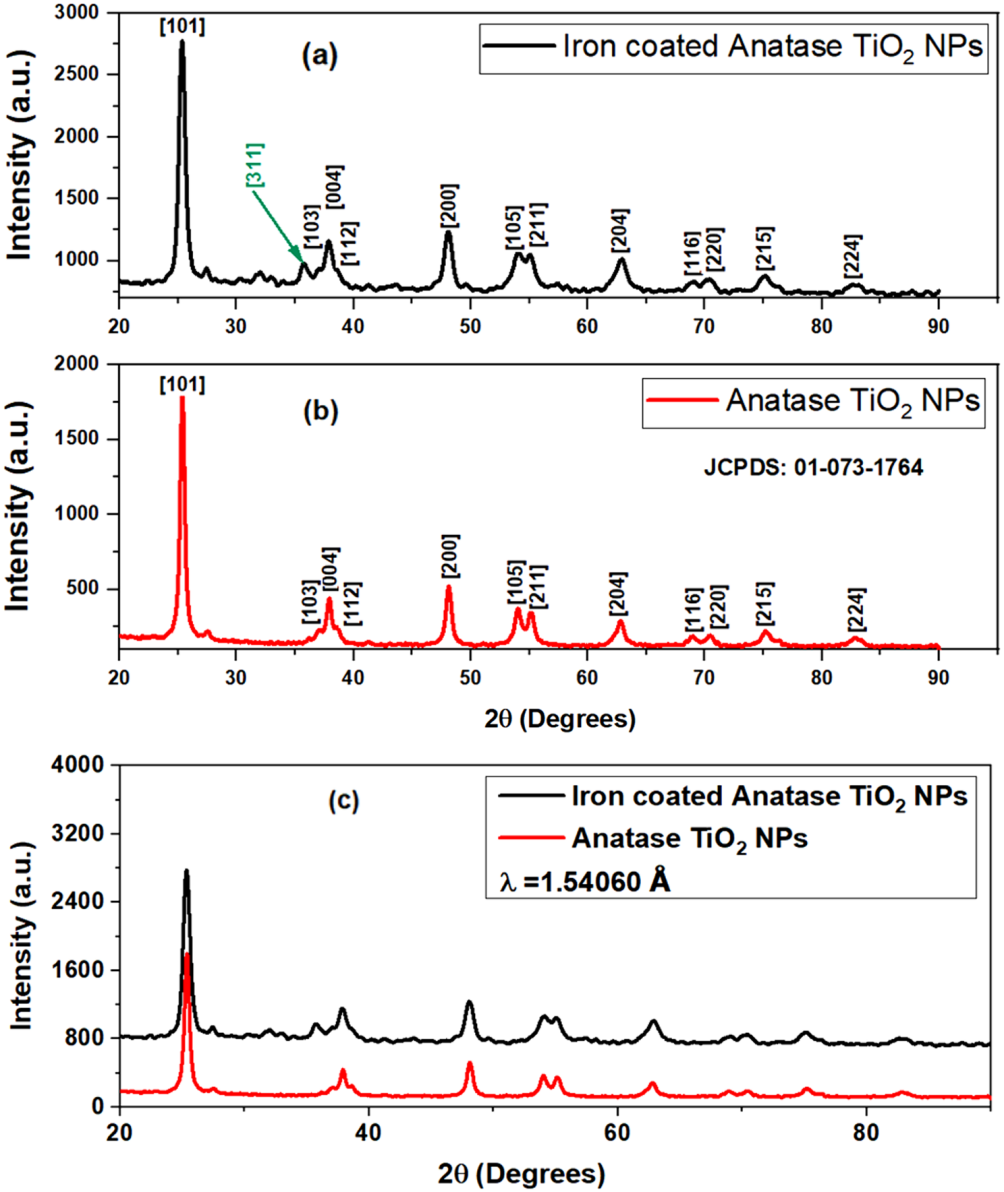
XRD pattern of (**a**) Fe@TiO_2_ NPs, (**b**) TiO_2_ NPs and (**c**) superimposed XRD plots obtained in (**a**) and (**b**).

The samples were subjected to Powder X-ray Diffraction using a PANalytical X-Pert Pro X-ray diffractometer having Cu as anode material and having K_α1_ as 1.54060 Å. Figure 2a depicts the XRD plot retrieved for Fe@TiO_2_ NPs and Fig. 2b shows the XRD pattern obtained for bare TiO_2_ NPs. Figure 2b showcases anatase TiO_2_ NPs obtained on calcination at 400 °C. The peak at 25.3764° confirms the [101] plane of the anatase phase. The entire pattern matched well with the JCPDS pattern: 01-073-1764 confirming the peaks of anatase TiO_2_ NPs. Different planes recognized from the plot are [101] at 25.322°, [103] at 36.998°, [004] at 37.863°, [112] at 38.600°, [200] at 48.064°, [105] at 53.975°, [211] at 55.094°, [204] at 62.756°, [116] at 68.870°, [220] at 70.330°, [215] at 75.141° and [224] at 82.756°. Fig. 2a demonstrates the XRD peaks of Fe@TiO_2_ NPs and here the iron-capping has been done on the same TiO_2_ NPs whose XRD obtained is shown in Fig. 2b. We clearly observe that both the patterns are identical and thus Fe@TiO_2_ NPs also possess the similar anatase phase, although the peaks are a little broadened. Whether the XRD pattern of the core–shell NPs will match with that of the core NPs, this depends upon the thickness of the shell. The possibility of Fe@TiO_2_ NPs having the similar XRD pattern can be attributed to the thin layer of coating on the TiO_2_ NPs. The incident x-rays can easily penetrate the shell thickness of ~10 nm and below and thus almost a similar pattern is obtained. Moreover, a careful investigation indicates that one extra peak has emerged at 35.631° which corresponds to plane [311] of cubic Fe_2_O_3_ as denoted by JCPDS pattern: 00-039-1346. This is justified as the coating of ferrous fumarate (Fe^2+^) was done onto the TiO_2_ NPs. The crystallite size calculated using Debye–Scherrer’s equation stated the size of ~12 nm for TiO_2_ NPs and ~17 nm for Fe@TiO_2_ NPs. Furthermore, Fig. 2c depicts the superimposed XRD patterns obtained in both Fig. 2a, b. One can easily tell that the entire XRD plot of Fe@TiO_2_ NPs has been shifted up in intensity. This is a striking observation that the core–shell Fe@TiO_2_ NPs retain the similar XRD pattern of the core TiO_2_ NPs. The basic idea of core–shell formulation is to have a uniform coating while still conserving the properties of the core. The layer formed must never be too thick; otherwise, it will overshadow the features of the core^25^.

**Figure 3:**
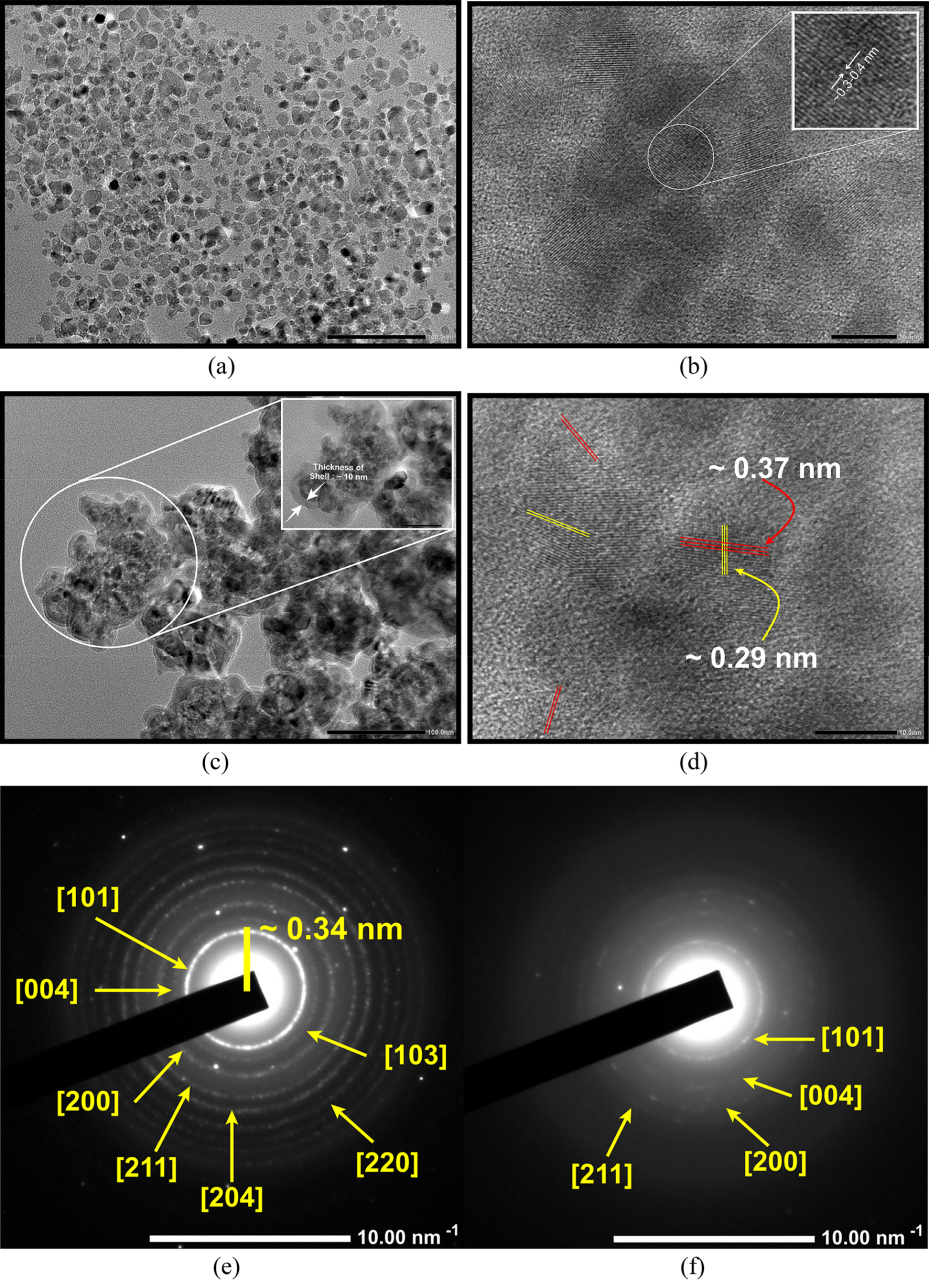
(**a**, **b**) HR-TEM images of TiO_2_ NPs, (**c**, **d**) HR-TEM images of Fe@TiO_2_ NPs, (**e**) SAED pattern of TiO_2_ NPs and (**f**) SAED pattern of Fe@TiO_2_ NPs.

The High-Resolution Transmission Electron Microscopy (HR-TEM) images of TiO_2_ NPs are shown in Fig. 3a, b whereas Fig. 3c, d demonstrate the HR-TEM images of Fe@TiO_2_ NPs. Figure 3a shows that the average size of the TiO_2_ NPs appear to be ~10 nm. This goes along with the crystallite size calculated from the respective XRD plot. The particles are nearly spherical and there is a uniform size distribution. The interplanar distance lies between 0.3 and 0.4 nm, more precisely 0.37 nm as observed in Fig. 3b. The inset shows the magnified view of the region selected to measure the interplanar distance. In Fig. 3c, we can clearly see the core–shell formulation of the NPs. Now, the NPs are no longer isolated, but agglomerated and an external layer/capping is distinctly visible. The darker portions indicate the presence of element with lower atomic number and here relate to the core i.e. TiO_2_ NPs. The lighter portions indicate the presence of an element with a higher atomic number which is Fe^2+^ form of the iron in this case and corresponds to the shell. The inset displays the magnified view of the image which has been used to measure the thickness of the layer. The scale of the inset is 50 nm and with this, it is deduced that the layer is at most 10 nm thick. In Fig. 3d, two different interatomic spacings of 0.37 nm and 0.29 nm can be seen overlapping in most of the regions. Figure 3e depicts the Selected Area Electron Diffraction (SAED) pattern for TiO_2_ NPs which matches well with the corresponding XRD pattern. Fully bright dots and distinct rings are clearly visible. This confirms the highly crystalline nature of TiO_2_ NPs. However, the SAED pattern for Fe@TiO_2_ NPs in Fig. 3f consists of diffuse rings which signifies their less crystalline nature. Once again, this can be confirmed from the respective XRD pattern in Fig. 2a. Thus, the findings obtained from XRD, HR-TEM and SAED analysis are in good agreement with each other.

**Figure 4:**
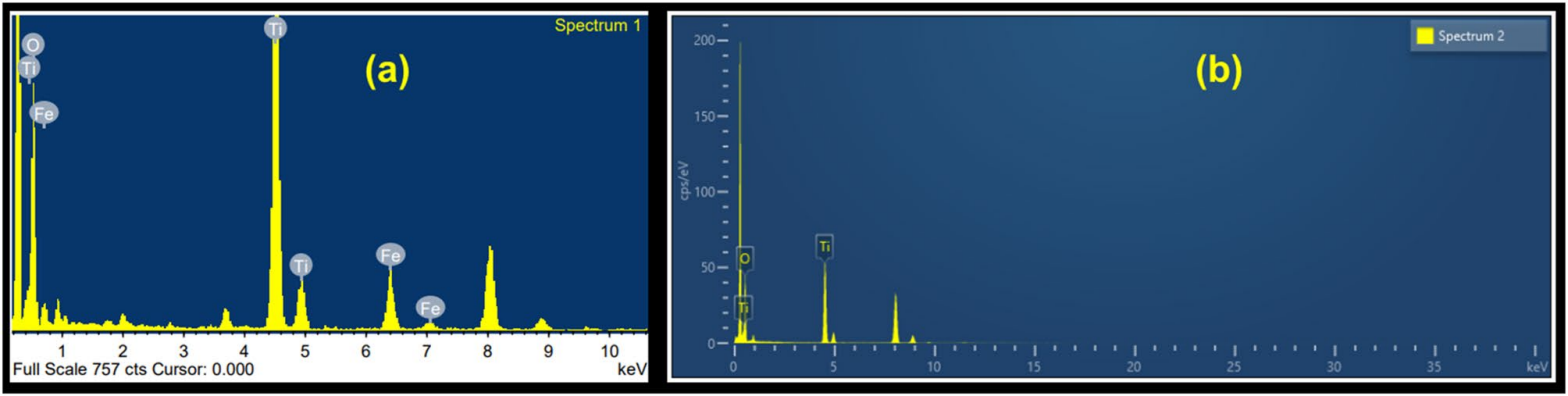
(**a**) EDX of Fe@TiO_2_ NPs and (**b**) EDX of TiO_2_ NPs.

**Figure 5:**
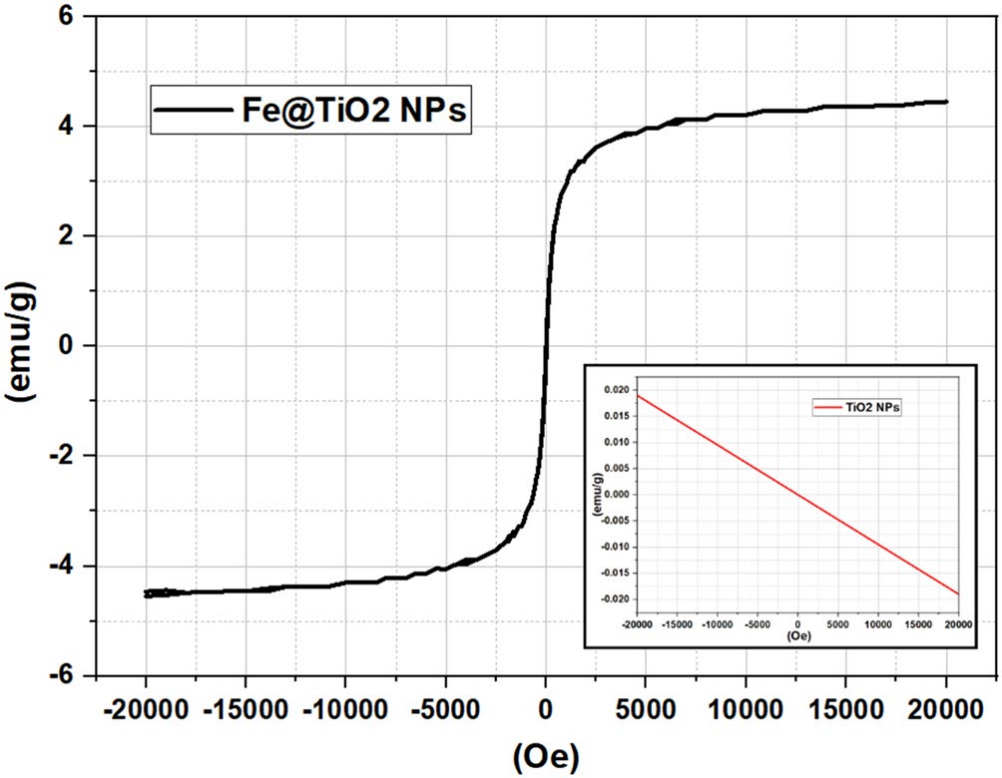
The VSM plot of Fe@TiO_2_ NPs depicts superparamagnetic behavior. The inset displays the diamagnetic character of pure anatase TiO_2_ NPs.

The Energy Dispersive X-Ray Analysis (referred to as EDX or EDS or sometimes EDAX) confirms the presence of elements Oxygen (O), Titanium (Ti) and Iron (Fe) in Fe@TiO_2_ NPs as shown in Fig. 4a. Similarly, Fig. 4b depicts the presence of Oxygen (O) and Titanium (Ti) in TiO_2_ NPs. Table 1 outlines the weight percentage and atomic percentage of all the elements in both the samples. The Vibrating Sample Magnetometer (VSM) studies of both Fe@TiO_2_ NPs and TiO_2_ NPs are shown in Fig. 5. The inset of Fig. 5 illustrates the pure diamagnetic nature of anatase TiO_2_ NPs. The room temperature magnetism induced for the applied magnetic field is a linear graph with a negative slope indicating diamagnetism for TiO_2_ NPs. The plot of the diamagnetic behavior is similar to the previously reported result^26^. However, the VSM plot of TiO_2_ NPs capped with an iron supplement capsule shows superparamagnetic behavior. Beketova et al.^27^ decorated TiO_2_ nanotubes with Fe_3_O_4_ NPs which also reported superparamagnetic behavior at room temperature. This is a first in itself effort to magnetically modify the NPs using commercially available iron supplements manufactured for human consumption. Thus, Fe@TiO_2_ NPs can be driven to the target sites with the help of external magnetic fields. Table 2 summarizes the hysteresis parameters of Fe@TiO_2_ NPs obtained via VSM against Magnetization (emu) vs Applied Magnetic Field (Oe).

**Table 1:** Weight percentage and atomic percentage of elemental composition of Fe@TiO_2_ NPs and TiO_2_ NPs.

Sample → Element ↓	Fe@TiO_2_ NPs		TiO_2_ NPs	
	Weight%	Atomic%	Weight%	Atomic%
O	30.42	57.20	39.18	65.86
Ti	59.58	37.41	60.82	34.14
Fe	10.00	5.38	–	–
Total	100	100	100	100

**Table 2:** Hysteresis parameters obtained for Fe@TiO_2_ NPs in the emu vs Oe plot.

Hysteresis loop	Upward part	Downward part	Average	Parameter ‘definition’
				Hysteresis parameters
H_c_ (Oe)	17.349	−7.774	12.561	Coercive field: field at which M/H changes sign
M_r_ (emu)	−4.827E−3	2.180E−3	3.503E−3	Remanent magnetization: M at H = 0
S	0.040	0.018	0.029	Squareness: M_r_/M_s_
S*	0.539	1.346	0.942	1 − (M_r_/H_c_)(1/Slope at H_c_)
M_s_ (emu)	120.250E−3	−122.678E−3	121.464E−3	Saturation magnetization: maximum M measured
M at H_max_ (emu)	120.250E−3	−122.678E−3	121.464E−3	M at the maximum field

For the drug release studies, we have investigated the release behavior under different pH levels. The following terminology has been used to indicate the samples in different pH solutions as shown in Table 3.

**Table 3:** Different terminologies used for samples under different pH levels.

pH → Samples ↓	pH 4.4	pH 7.4	pH 9.0
TiO_2_ NPs	T4	T7	T9
Fe@TiO_2_ NPs	F4	F7	F9

**Figure 6:**
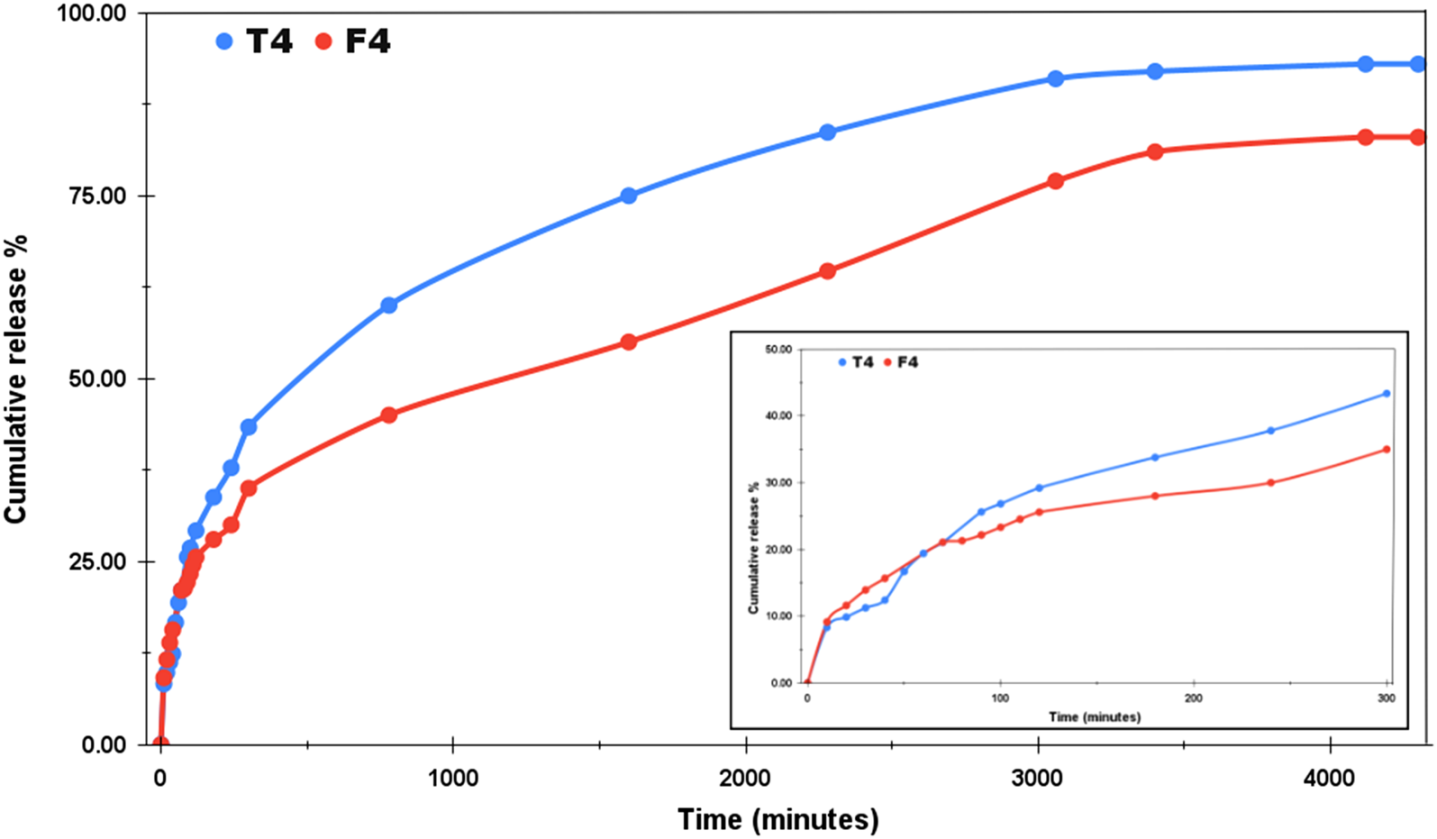
The Imatinib release profiles of Drug/TiO_2_ NPs and Drug/Fe@TiO_2_ NPs at pH 4.4.

**Figure 7:**
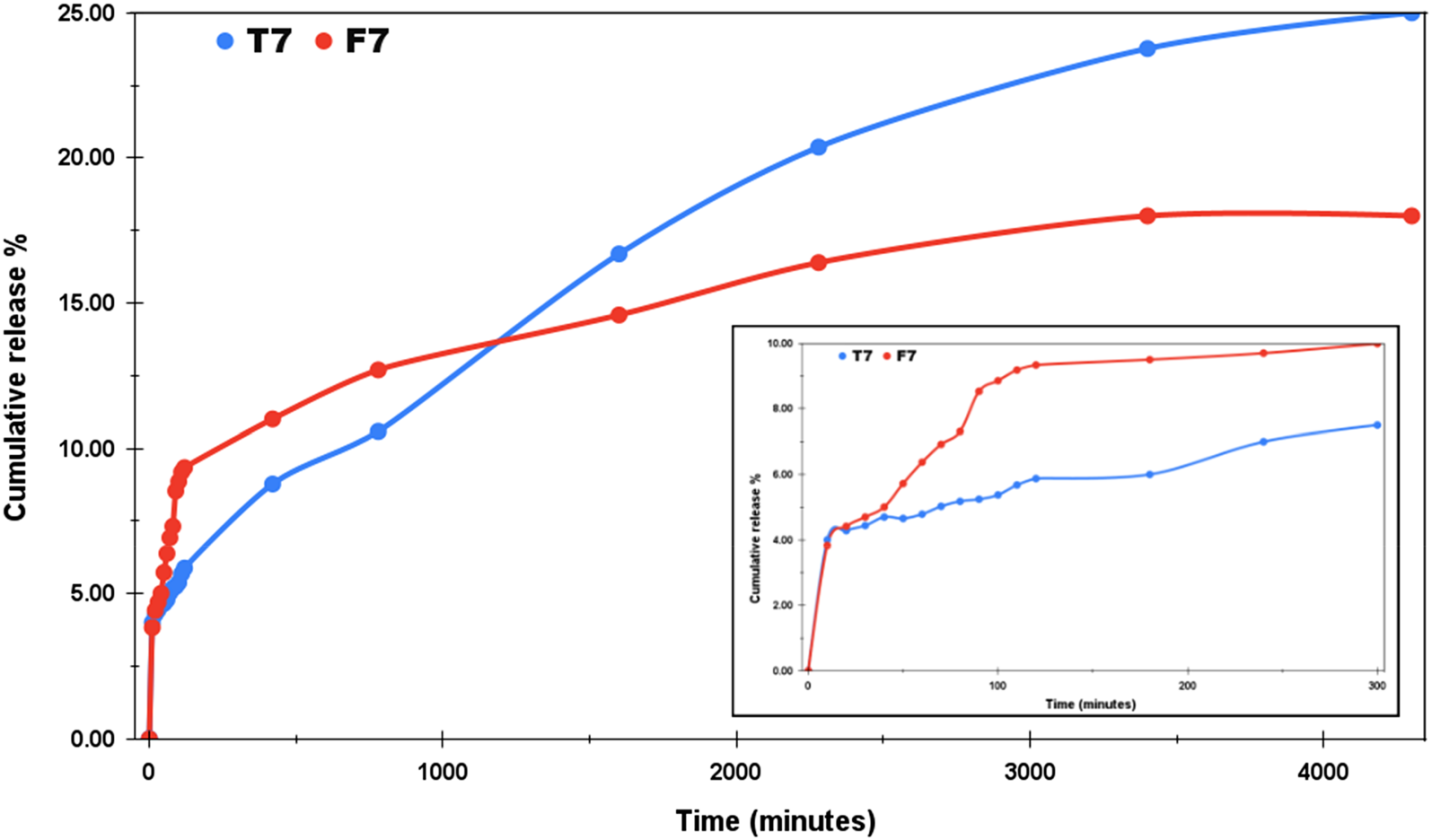
The Imatinib release profiles of Drug/TiO_2_ NPs and Drug/Fe@TiO_2_ NPs at pH 7.4.

**Figure 8:**
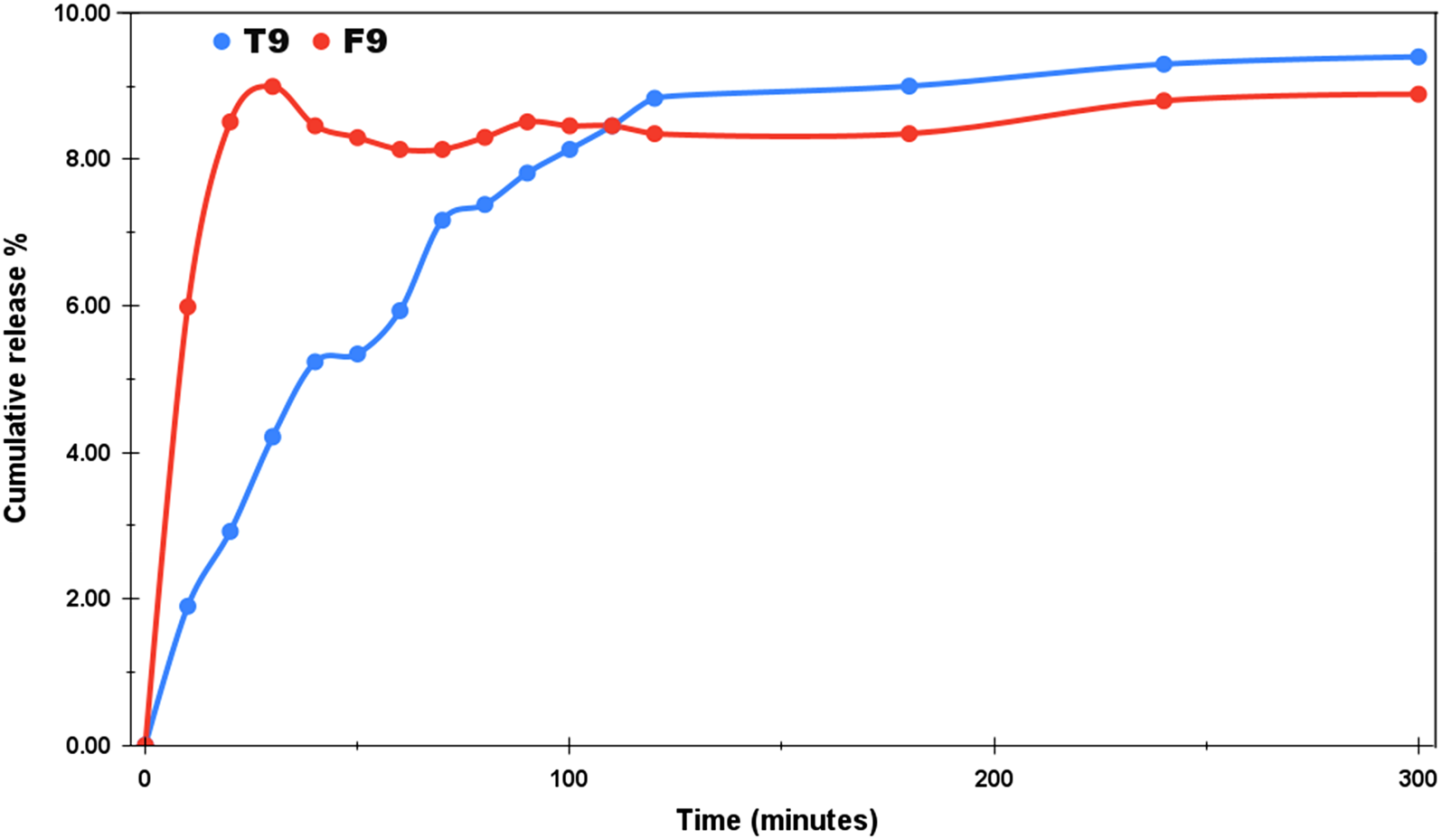
The Imatinib release profiles of Drug/TiO_2_ NPs and Drug/Fe@TiO_2_ NPs at pH 9.0.

For both the acidic and the normal mediums, the study was undertaken for three days. The choice of dialysis tube also affects the pattern of drug release. Figure 6 represents the drug release profile of T4 and F4 at pH 4.4 with cumulative release percentage plotted along the y-axis and the time (in minutes) along the x-axis. The pH around carcinogenic tumors is usually acidic, that’s why most of the chemo drugs are designed to deliver faster around acidic environments. Moreover, the orally administered drugs reach first into the stomach after consumption, where the highly acidic medium breaks it down into its components from where the molecules enter into the bloodstream. In our case, the initial release in the first two hours was much faster as compared to the total study of 3 days. T4 and F4 released upto 29.2% and 25.58% respectively at the end of 120 min. T4 showed the cumulative release of 60% in 14 h and finally reached the maximum release of 93% at the end of our study. F4 displayed a rather controlled release pattern presenting approx. 50% cumulative release after 26 h. The maximum release % recorded for F4 at the end of three days is 83%. The inset in Fig. 6 provides the drug release pattern for the first 300 min. Thus, even under the acidic medium, both T4 and F4 showed a sustained release pattern and this attribute can be utilized for the controlled delivery of the drug. Likewise, Fig. 7 demonstrates the drug release profile of T7 and F7 at pH 7.4. The pH of normal blood is generally considered to be between the range 7.35–7.45. The NPs to be used as drug carriers are normally expected to exhibit controlled but faster drug release under acidic environments and less to no release under normal pH. By this we can assure lesser drug wastage during the circulation and more effective drug release at the target locations. Under normal pH, we can see that in the span of three days, the maximum cumulative release percentage was 25% and 18% for T7 and F7 respectively. No further release was observed and F7 has outperformed T7 in the context of least drug release in the normal pH. The inset in Fig. 7 shows the initial release pattern in the first 300 min. T7 presented 5.87% release and F7 showed 9.34% cumulative release in the initial 300 min. Furthermore, the samples were also tested under the basic medium of pH 9 and as expected, an overall release of 9.4% was observed for T9 and 8.9% was observed for F9 as shown in Fig. 8. This study was only stretched for 300 min as no appreciable release was seen after 120 min. This study confirms that the drug release rate of both TiO_2_ NPs and Fe@TiO_2_ NPs is pH-dependent. The maximum release has been detected in the acidic medium and the least release in the basic medium. Thus, both types of NPs are suitable candidates to be used as drug-delivery agents. Fe@TiO_2_ NPs exhibited a more controlled drug-release behavior and therefore, the maximal release attained by them is less than TiO_2_ NPs under all the pH levels.

In-vitro study conducted by Kadivar et al.^28^ suggests that 99.56% of the Imatinib is released within 20 min in 0.1 N HCl solution. In comparison to this observation, both the NPs synthesized by us demonstrate much controlled and sustained release of the drug (Imatinib) which is very beneficial for an effective chemotherapeutic treatment. If the drug is retained for a longer time, this would minimize the drug dosages required which would further control the side-effects. The results of the in-vitro study revealed the attainment of maximum drug release under an acidic environment by these NPs, suggesting that they undergo relatively faster decomposition thereby promoting faster release at low pH level than at neutral pH level. CaCO_3_ NPs demonstrate similar pH-responsive behaviour and have been extensively studied for drug delivery applications. They exhibit low toxicity, slow biodegradability and when functionalized, they not only augment the effectiveness of drug-delivery, but also enhance drug-loading, tumor-targeting and thermodynamic stability^29^. Thus, NPs need to be improved for showcasing multiple benefits.

**Figure 9:**
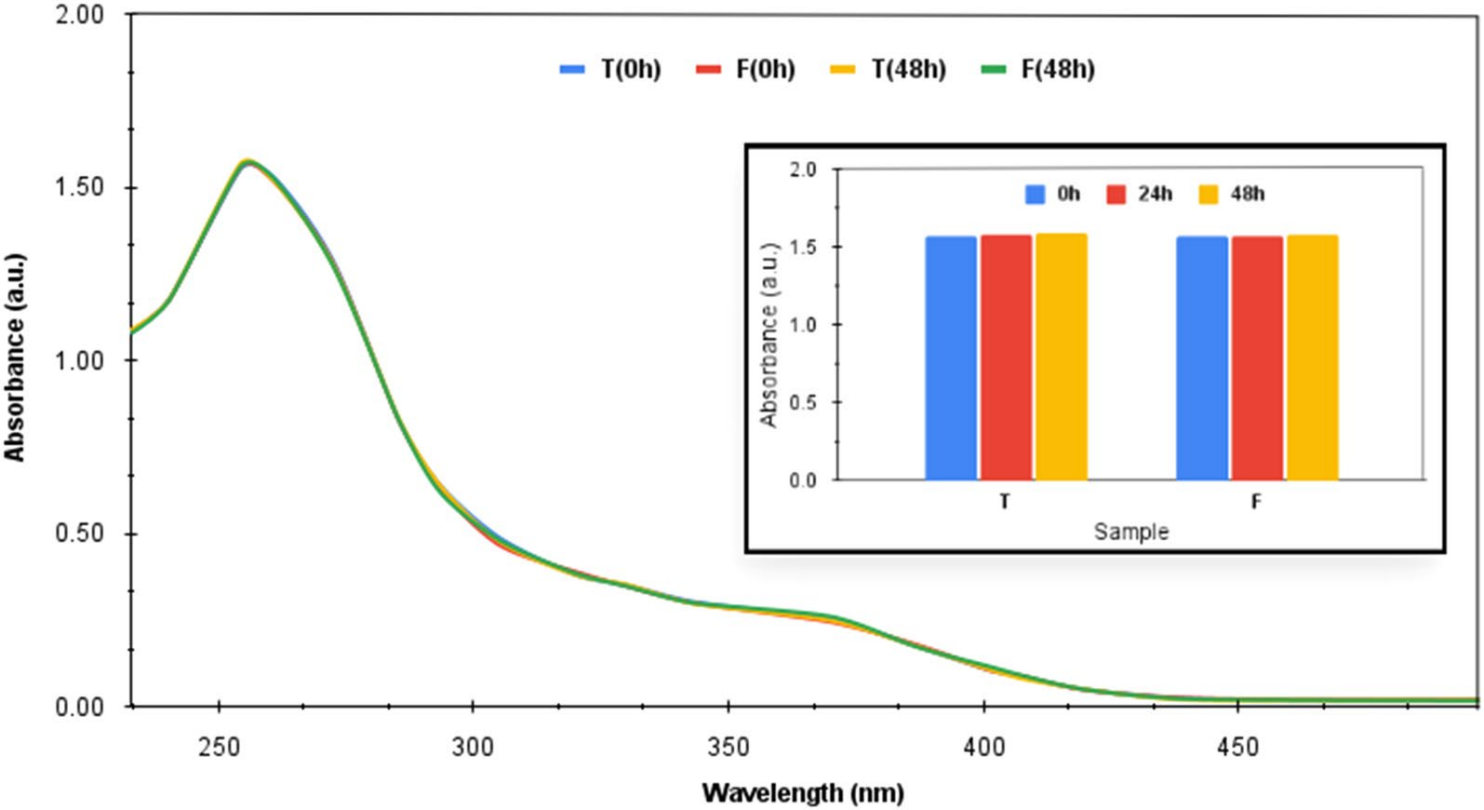
UV–visible absorbance plot at different time intervals for incubated NBT solution along with TiO_2_ NPs and Fe@TiO_2_ NPs separately. The inset compares the individual performances of TiO_2_ NPs and Fe@TiO_2_ NPs over the period of time.

Figure 9 showcases the UV–visible plots for both the NPs taken at 0 h i.e. at the beginning of the study and at 48 h (at the end of the study), respectively. ‘T’ represents the TiO_2_ NPs and ‘F’ represents the Fe@TiO_2_ NPs. The inset of Fig. 9 shows the comparison of the absorbance of the NBT solution recorded at 254 nm over three different intervals of time i.e. 0 h, 24 h and 48 h respectively in the presence of both the NPs separately. It is observable that there was no significant decrease in the absorbance of the characteristic peak at 254 nm over the period of time. The plots of TiO_2_ NPs-NBT solution and Fe@TiO_2_ NPs-NBT solution taken over different instants of time almost overlapped. Moreover, no colour change was detected by observing the samples with naked eye. Thus, the ROS detection test conducted by us for both the NPs validate that no ROS is generated by these NPs under human physiological conditions which confirms their biosafety. Basante-Romo et al.^30^ modified TiO_2_ NPs with functionalized multiwalled carbon nanotubes and evaluated them for their level of toxicity. The toxicity studies were performed on female albino rats and no adverse effects were observed after the 10-days study. No mortality was produced. All the clinical signs were normal suggesting that no changes occurred in the cells or tissues exhibiting normal architecture of the organs. Thus, the modification of TiO_2_ NPs diminishes the chances of them being destructive. The coating of these NPs with a biocompatible material and other sorts of surface modifications assure that these TiO_2_ NPs won't be displaying their ROS generation ability sans irradiation. Since these NPs are not irradiated when used for drug-delivery applications, the chances of them resulting in lethal ROS are very low.

### Future perspective

The usage of NPs as drug-delivery agents belong to new-era medical techniques. A lot of research is going on to ensure the suitability of these NPs for the said purpose. This very idea is still in its infancy and it has to successfully pass a plenty of pre-clinical and clinical trials to be actually able for a certified medical usage. Talking about ensuring targeted drug delivery of chemotherapeutics, emphasis should be laid on a variety of factors including the mitochondrial response, the oxidative stress, the receptors present, any sort of inherent or induced drug resistant response of the cellular components and many more. Furthermore, any kind of toxicity offered by the NPs should also be dealt with great care. The toxicity of the NPs does not lie only in the formation of ROS, but the size and shape of the NPs are also the factors of grave concern. The NPs should be optimally synthesized to have optimal size and shape which offer least accumulation in the tissues and organs. Different techniques to functionalize the NPs, to modify their surface properties, to optimize their size and shape, to ensure adequate amount of drug loading onto them, to improve their antioxidant characteristics and to make them biocompatible, should be devised. Thus, adequate steps should be taken to ensure the biosafety of the NPs to be used as drug-carriers and to further improve their drug-delivery response. Once satisfactory results are achieved from in-vitro studies, only then more systematic approaches can be explored to guide in-vivo research and better correlate the properties of nanoparticles with their biological effects.

## Conclusion

Anatase TiO_2_ NPs and Fe@TiO_2_ NPs were successfully synthesized and their properties were assessed by different characterization techniques including XRD, HR-TEM, SAED, EDX and VSM. To induce magnetic behavior in TiO_2_ NPs, locally available iron supplements were used to synthesize Fe@TiO_2_ NPs which displayed magnetic responsiveness. The amount of drug released was the highest under acidic pH among the different pH levels chosen for the study. The drug-nanocarrier formulations exhibited much sustained drug release for a prolonged period of time which is much better than the 20-min quick release of the drug alone under acidic conditions. In addition to this, Fe@TiO_2_ NPs can be used productively for the targeted drug delivery with the help of external magnetic fields in the treatment of carcinogenic tissues. The ROS detection test conducted in this study has also given clean chit to these NPs on the grounds of biosafety offered by them. On the basis of the current work, pH-responsive NPs based drug delivery systems might be able to replace the traditional chemotherapy systems and eliminate its side-effects. However, this study deals with just one aspect of the drug-release mechanism which is pH-responsive. Further modifications can be done to the NPs to improve their biocompatibility, their encapsulation efficiency and % drug loading. The future aspects lie in in-vivo investigations of the formulations discussed in the present study.
